# Antiparasitic activity of the iron-containing milk protein lactoferrin and its potential derivatives against human intestinal and blood parasites

**DOI:** 10.3389/fpara.2023.1330398

**Published:** 2024-02-28

**Authors:** Namrata Anand

**Affiliations:** Department of Pharmacy Practice and Science, College of Pharmacy, University of Kentucky, Lexington, KY, United States

**Keywords:** parasites, lactoferrin (Lf), bovine lactoferrin (bLf), human lactoferrin (hLf), iron chelation, Lf peptide, Lf nanoformulation

## Abstract

An iron-containing milk protein named lactoferrin (Lf) has demonstrated antiparasitic and immunomodulatory properties against a variety of human parasites. This protein has shown its capability to bind and transport iron molecules in the vicinity of the host–pathogen environment. The ability of parasites to sequester the iron molecule and to increase their pathogenicity and survival depends on the availability of iron sources. Lf protein has suggested a iron chelating effect on parasites iron and, hence, has shown its antiparasitic effect. Since the parasites have a complex life cycle and have developed drug resistance, vaccines and other treatments are a handful. Therefore, therapeutic research focusing on natural treatment regimens that target the parasite and are non-toxic to host cells is urgently needed. The antiparasitic efficacy of Lf protein has been extensively studied over the past 40 years using both *in vitro* and *in vivo* studies. This review article highlighted past important studies on Lf protein that revealed its potential antiparasitic activity against various intracellular and extracellular intestinal or blood-borne human parasites. This review article structures the role of Lf protein in its various forms, such as native, peptide, and nanoformulation, laying the groundwork for its function as an antiparasitic agent and its possible known mechanisms of action.

## 1 Introduction

Parasites are responsible for many human diseases worldwide. One of the most life-threatening parasitic infections is malaria, which was responsible for 247 million cases and more than 600,000 deaths in the year 2022 (WHO, 2022). Some other blood-borne parasitic infections, such as trypanosomiasis, leishmaniasis, toxoplasmosis, and filariasis, are also responsible for millions of cases each year and have a high disease burden (Drame et al., 2016; Theel and Pritt, 2016). Intestinal infections such as giardiasis, amoebiasis, cryptosporidiosis, and helminthic infection are more common in tropical and non-tropical regions (Suntaravitun and Dokmaikaw, 2018; Chen et al., 2022; Nath et al., 2022). The incidence rate is high in low income countries and areas of poor sanitation (Kamau et al., 2012; Hajare et al., 2021). Parasites cause zoonotic infections that transmit between vertebrates and invertebrate hosts and involve multiple life cycle stages. Interruption in transmission in different stages and hosts may lead to their mortality (Auld and Tinsley, 2015). Multiple life cycle stages in different hosts leads to treatment failure and drug resistance, which has been known in many parasites. *Plasmodium* has shown drug resistance to conventional drugs such as chloroquine, mefloquine, and artemisinin (Shibeshi et al., 2020). Artemisinin combination therapy (ACT) has also shown emerging resistance since 2008 in South Asian countries, which continues to spread (Kagoro et al., 2022). *Leishmania* parasite has also shown drug resistance towards pentavalent antimonial and pentamidines (Maltezou, 2010). Amphotericin B has been the second line of treatment against leishmaniasis and has shown effective results, but because of its numerous off-target toxicities, its usage is limited (Laniado-Laborín and Cabrales-Vargas, 2009). There are multiple drugs or secondary metabolites used to target human and animal protozoan and helminthic parasites to control the spread of disease (Velázquez-Antunez et al., 2023). Many studies have suggested the role of plant-based therapy against blood-borne parasites, which are under development and further require *in vivo* studies (Kaur et al., 2021; Qureshi, 2021; Kamaraj et al., 2022; Benlarbi et al., 2023; Wulandari et al., 2023). Apart from drug treatment strategies, various preventive measures are required to control the spread of these zoonotic infections worldwide (Molyneux, 2006; Ghodsi et al., 2019; Luan et al., 2023).

Iron is one of the most important nutrient elements that help humans and other organisms grow. It is also the most important element responsible for host–pathogen interaction as it involves various cellular and metabolic processes. Previous studies have found an association between parasitic infections and low iron anemia in children (Osazuwa et al., 2011; Arroyo et al., 2015; Gujo and Kare, 2021; Kaur and Juneja, 2022). Various parasites sequester the iron present at the cellular level through proteins such as transferrin (Tf), lactoferrin (Lf), hemoglobin (Hb), and other iron pools (Collins, 2003). These proteins are the main source of Fe for intracellular pathogens in macrophages and red blood cells (RBCs), and for extracellular pathogens, epithelial cells act as an iron source. Upon pathogenic attack, Fe is released in the form of degraded cell components or degradation of RBCs, and parasites acquire this iron for their growth and multiplication. Availability of this iron may be diminished if it can be sequestered and if it is not available for pathogens for their multiplication, pathogenesis, or growth (Skaar, 2010).

Lf is a multifunctional glycoprotein, belonging to the family of Tf, which helps in the binding and transferring of iron (Fe^3+^) molecules and acts as an iron sequester. It was first isolated in 1939 and identified as a red protein from bovine milk (Sorensen, 1939). The three-dimensional structure of Lf was identified in 1987, the first from its family to be identified (Anderson et al., 1987). These proteins are found in mammals and are involved in providing defense against various microbial infections and also act as an essential growth factor (Naot et al., 2005; Siqueiros-Cendón et al., 2014). Lf is predominantly present in mammary glands, in secretions of exocrine glands, and in specific granules of neutrophils, reaching a concentration as high as 8 mg/mL. The major source of Lf present in the blood is derived from neutrophil degranulation (Lepanto et al., 2019). All of these proteins possess a common structural pattern, consisting of a single polypeptide chain of approximately 700 amino acid residues with two homologs, N- and C-terminal lobes of a similar length (Baker and Baker, 2009). These two lobes and four domain structures provide the basic functional understanding of the protein. Each lobe binds reversibly to two iron (Fe^3+^) and together with carbonate ions (CO_2_^3−^) (Legrand et al., 2008).

There are three different structural forms of Lf according to its iron saturation: apo Lf (no iron), mono Lf (one iron), and holo Lf (two iron), which provide its three different dimensional structures (Baker and Baker, 2009). Besides Fe^3+^, Lf protein is also capable of binding to lipopolysaccharides (LPS), heparin, DNA, and metal ions such as Al^3+^, Mn^3+^, Co^3+^, Cu^2+^, and Zn^2+^, which express its antimicrobial ability with diverse modes of action (Honarparvar et al., 2019; Rogowska et al., 2023). Holo Lf gives it the most packed and stable structure, with two Fe^3+^ that synergistically bind two CO_2_^3−^ ions (Baker and Baker, 2009). This form of Lf allows unrestricted access for binding with the neighboring iron molecule and various other metal ions. The apo Lf is much less stable and less compact and is therefore prone to heat denaturation and proteolysis (Baker and Baker, 2004). The release of iron from holo Lf results in its destabilization, which could be triggered by lowering the pH to 4. Therefore, depending on the presence of one or two iron molecules or its absence, Lf protein can donate/deprive or sequester the iron molecule and hence can perform antitumor, antiparasitic, antibacterial, antifungal, or antiviral activities, along with immunomodulation (Hao et al., 2019; Ahmed et al., 2021). There are many review articles that have discussed the antimicrobial activity of Lf protein, but there are a handful of publications that have suggested Lf protein as an antiparasitic agent in its different formulation. This review article highlights the importance of this novel drug molecule that can be used in its various derivations, like its peptide form, and can be incorporated in nanoformulations to target parasitic infections.

## 2 Effect of Lf protein on intestinal parasites

This section discusses important studies of human intestinal parasites such as *Entamoeba*, *Giardia*, and *Cryptosporidium*, which are known to be vulnerable to the treatment of Lf protein and its peptides. Different forms of Lf studied against intestinal parasites with various sources of origin, target stages, and concentrations are summarized in **Table 1**.

**Table 1:** Antiparasitic activity of various Lf forms against intestinal parasites.

Parasite Stage	Lactoferrin source and concentration used		Percentage Viability	Lactoferrin Peptide used	Percentage Viability
***Entamoeba*** Trophozoite	Human holo Lf	(50µM) Leon-Sicairos et al., 2005)	93%	Lfchimera (100µM)(López-Soto et al., 2010)	>5-6%
			52%	Lfamphin (500µM)(Díaz-Godínez et al., 2019)	10%
	Human apo Lf	(1mg/ml) (Leon-Sicairos, Lopez-Soto et al. 2006)			
	Bovine apo Lf	(1mg/ml) (León-Sicairos, López-Soto et al. 2006)	66%		
***Giardia*** Trophozoite	HLf (2.5.mg/ml)(Turchany et al., 1995)		25%	HLfcin (24µg/ml)(Turchany et al., 1995)	25%
	BLf (2.0 mg/ml)(Turchany et al., 1995)		20%	BLfcin (12 µg/ml)(Turchany et al., 1995)	22%
	BLf apo (12.5µM)(Turchany et al., 1995)		~25%		
				Lfcin, Lf chimera, Lf ampin (40 µM) (Aguilar-Diaz et al., 2017)	42%,25%,45%
				Lfcin (2.6 µM)(Frontera et al., 2018)	~50%
***Cryptosporidium*** Sporozoite	bLf (10µg/ml)(Carryn et al., 2012)		90%	Lf hydrolysate(Carryn et al., 2012)	55%,
				Lfcin B (10µg/ml)(Carryn et al., 2012)	50%

### 2.1 Endocytosis of holo Lf protecting *Entamoeba histolytica* trophozoite and apo Lf depleting its nutrients

*Entamoeba histolytica* (*E. histolytica*) is a protozoan parasite and the causative agent of amoebiasis in humans causing 500 million cases and approximately 100,000 deaths annually. The disease is transmitted through parasitic cysts that are ingested through contaminated food and water, thus increasing the incidence of this disease in areas with poor sanitation (Nasrallah et al., 2022). Amoeba requires iron for its growth and obtain it by engulfing bacteria and RBCs that help increase its iron source through its endocytic pathway. A previous study has suggested the possible interaction between the amoebic parasite with bovine Lf (bLf) and human Lf (hLf). It was theorized that parasites takes up Lf iron through membrane internalization and tubular invaginations (de JO Batista et al., 2000; Leon-Sicairos et al., 2005; López-Soto et al., 2009). Further potential evidence suggested the amoebicidal activity of the apo form of Lf present in human and bovine milk and tested at the trophozite stage. Effect was seen through iron sequestration via receptor-mediated binding and depriving its essential nutrient source (Leon-Sicairos et al., 2006a). This anti-amoebic activity of Lf was further supported by an *in vivo* study where infected C3H/HeJ mice were orally administered with bLf and post-treatment showed a significantly increased Th1 type of immune response and reduced swelling in the cecum with no histopathological damage to the intestinal tissue (León-Sicairos et al., 2012). These findings were further confirmed using the hamster model of amoebic liver abscess, and upon treatment, liver abscess was found to heal completely and showed a high blood cell count and normal liver function (Ordaz-Pichardo et al., 2012).

### 2.2 Anti-amoebic and immunomodulatory effects of Lf peptides

Different antimicrobial peptides (AMPs) derived from hLf and bLf have been used against intestinal parasites. Lactoferricin B (Lfcin B) obtained from calf rennet hydrolysate Lf (4–14) and Lfcin (17–30) obtained after cleavage with pepsin showed inhibition towards the trophozoite stage of *E. histolytica*. Lfcin showed a synergistic effect with metronidazole against the amoebic parasite when observed in a time- and dose-dependent manner (Leon-Sicairos et al., 2006b) (**Table 1**). Other potent peptides, namely, Lfampin (265–284) and Lfchimera, which is composed of Lfcin and Lfamphin with a lysin link, were also found to be amoebicidal in nature and showed the highest parasiticidal activity among the tested compounds (López-Soto et al., 2010). A previous study has demonstrated the significant effect of Lfamphin peptide on *E. histolytica* and has demonstrated its receptor-mediated internalization in the trophozoite, through the P13K signaling pathway (Díaz-Godínez et al., 2019). The internalization of peptide induced changes in the actin cytoskeleton and its remodeling resulted in necrosis the parasite. The anti-amoebic effect of this peptide was later revealed in C3H/HeJ mice, which showed a complete absence of parasite infection in cecum tissue. The authors postulate the immunomodulatory effect of the Lfamphin peptide on mice, which helped their prolonged survival (Díaz-Godínez et al., 2019). These studies suggested the use of a small peptide sequence of Lf protein to act specifically in binding and its functional aspects over the native form of Lf.

### 2.3 Antigiardial activity of apo Lf protein against *Giardia*

*Giardia* is an intestinal parasite known to cause acute and chronic diarrhea, especially in children, and causes symptomatic infection in almost 280 million people annually, out of which 200 million are from Asia, Africa, and Latin America (Feng and Xiao, 2011). The most effective drugs against giardiasis are metronidazole, ornidazole, and tinidazole, showing a recovery rate of up to 90% (Escobedo and Cimerman, 2007; Vivancos et al., 2018). *Giardia* infection is more prone in iron-deficient children and induces iron deficiency anemia (IDA) and malabsorption (Gardner and Hill, 2001; Hussein et al., 2016). Because of the immunomodulatory activity of human breast milk and the presence of Lf protein, its efficacy was tested against *Giardia* trophozoites, which showed its potent antigiardial property and mitigating effect in children. These findings for the first time highlighted the potential role of human milk and presence of apo Lf causing the iron chelating effect on the parasite trophozoite (Breakey et al., 2015).

### 2.4 Antigiardial activity of Lf peptides on the trophozoite stage

Peptides derived from the N terminal of hLf (18 to 40) and bLf (17 to 41) peptides are the most potent AMPs that have demonstrated antimicrobial effects (Bellamy et al., 1992; Tomita et al., 1994). The N-terminal peptide derived from bLf has previously shown antigiardial activity when used at significantly low concentrations as compared with native form (**Table 1**). This peptide showed direct interaction with the trophozoite of the parasite, resulting in its killing effect (Turchany et al., 1995). Previous studies described the Lfchimera peptide as superior to the standard metronidazole drug when used in a dose- and time-dependent manner (Aguilar-Diaz et al., 2017). Treatment with Lfchimera led to morphological changes in the trophozoite such as flagellar disruption and distorted peripheral vacuoles, leading to apoptosis or programmed cell death (Aguilar-Diaz et al., 2017). Detailed ultra-structural examination on the *Giardia* trophozoite illustrated the direct interaction of native Lf and its peptide on the surface of the trophozoites, leading to deformation via flagellar swelling, disruption in plasma membrane, and ultimately, lysis of the cell (Turchany et al., 1997). Another study further illustrated the detailed mechanism of action of bLfcin against *G. lamblia*. *Giardia* trophozoites showed internalization of low-density lipoprotein (LDL) and chylomicrons through receptor-mediated engulfment (RME), and a similar pathway has been suggested by the authors for the engulfment of bLf and bLfcin. The authors suggested that this mechanism is essential for morphometric changes and lysis of the parasite, which was not known before (Frontera et al., 2018).

### 2.5 Antiparasitic effect of Lf and its peptides against *Cryptosporidium*

*Cryptosporidium parvum* (Ferrer et al., 2015; Thipubon et al., 2015) is responsible for the disease cryptosporidiosis, which causes intestinal infection in humans and animals, with an incidence rate of 1%–3% in Europe and North America and can also infect extra-intestinal organs in the case of an immunocompromised host (Hunter and Nichols, 2002; Semenza and Nichols, 2007; Ghallab et al., 2022), whereas *C. hominis* is only known to infect humans, but due to the lack of laboratory models to propagate *C. hominis* culture, most of the *in vitro* and *in vivo* studies have been performed with *C. parvum* (Gerace et al., 2019). Antibiotics such as nitazoxanide are used as the first line of treatment against *C. parvum*, but the results are not satisfactory and the mode of action of the drug is also not fully understood (Rossignol et al., 2006). Lf protein and its peptides, namely, Lfcin B and Lf hydrolysate (LfH), have previously demonstrated antiparasitic activity against *C. parvum* sporozoites with the highest inhibition rate achieved by LfH. This peptide also showed antiparasitic activity when used in combination with *Cryptosporidium* monoclonal antibody (3E2 MAb). The 3E2 MAb has been raised against the apical complex and surface glycoprotein ligand (CSL) of the parasite, which helps the parasite attach to the host cell. The combination of Mab and the Lf peptide helped inhibit sporozoite viability and infectivity (Carryn et al., 2012). Previous studies also examined the role of hLf on the excystation efficacy of *Cryptosporidium*. The authors showed possible inhibition of sporozoite viability and infectivity, but no effect was found on the excystation stage of the oocyst wall. These studies suggested that Lf protein or its peptides are impermeable to the thick wall of the oocyst but can inhibit the viability of free forms of this parasite through internalization via the endocytic pathway (Paredes et al., 2017).

## 3 Effect of Lf protein on intracellular blood parasites

**Figure 1:**
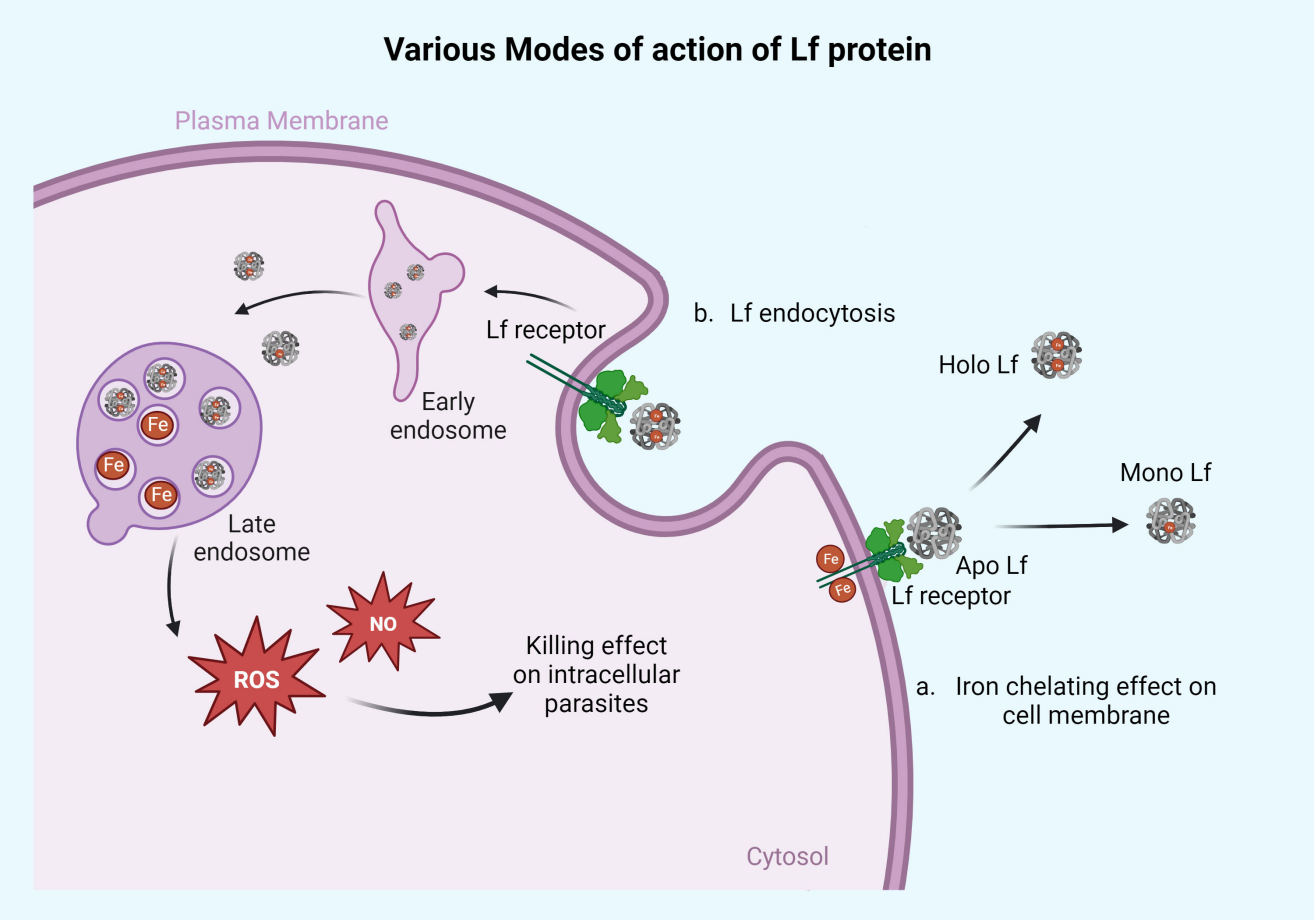
Action of Lf protein on the surface membrane of the parasites and intracellularly. Lf protein mainly acts on the parasites in two different ways. **(a)** Iron-chelating effect on cell membrane: the apo form of Lf chelates the Fe molecule present on the parasite surface membrane and converts it into the mono or holo Lf form. This way, apo Lf deprives the parasite of its iron source. This mechanism of action can be seen in parasites that reside/adhere extracellularly on the cell membrane of the host like *Giardia, Trichomonas*, and *Entamoeba.* The other part of the mechanism is based on Lf engulfment or through endocytosis, as seen in panel **(b)**. The Lf gets endocytosed and diffused with early and late endosomes, releasing the iron molecule into the cytoplasm, which causes free radical ion production in the form of ROS and NO leading to cell death of parasites. This high ROS or NO killing effect can be seen in parasites like *Leishmania, Plasmodium, Toxoplasma, Babesia*, and Trypanosoma. Retrieved from https://app.biorender.com/biorender-templates, December 2023.

Intracellular blood parasites such as *Leishmania, Plasmodium, Toxoplasma*, *Trypanosoma*, and *Babesia*, which are responsible for blood-borne infections, have been reported to be susceptible to the treatment of Lf protein. The apo/mono form of the Lf has been documented to show significant antiparasitic activity against these parasites by chelating the iron molecule with the help of the Lf receptor present on the parasite membrane (**Figure 1**), whereas holo Lf has shown support for their multiplication through internalization via endocytosis and further multiplication. Also holo Lf may lead to increased oxidative stress and kill the parasite. Various peptides and nanoformulations of Lf, along with its native form, have been used in studies and are summarized in **Table 2**.

**Table 2:** Antiparasitic activity of various Lf formulations against human blood parasites.

Parasite	Source of Lactoferrin and concentration used	Percentage Viability	Peptide concentration used	Percentag Viability	Lactoferrin Nanotherapy	Concentration of Lf used
***Leishmania*** Promastigotes and amastigotes	Bovine Lf peptides	50%(Silva et al., 2012)	Lfcin (22µM)	50%	AmpB PLGA nanoreservoir Betulinic Acid PLGA nanoparticles	0.03-0.6% (w/v)(Asthana et al., 2015) 1.5mg/ml (Halder et al., 2018)
			Lfchimera (4µM)	50%		
			Lfampin (31 µM)	50%		
***Plasmodium*** Blood Stage Merozoites	Human apo, holo Lf (30 µM) (Fritsch, et al., 1987)	Growth arrest in ring stage only	–	–	Alginate chitosan calcium phosphate calcium phosphate nanoparticles (AEC-CCO-CP-NC)	1.2% (w/v) (Anand et al., 2016)
*Spoprozoite*	Bovine Lf (Shakibaei and Frevert 1996)	89%	-	–	–	–
*Sporpzoite*	Human Lf (500µg/ml) (Sinnis et al., 1996)	74%	–	–	–	--
Blood Stage Merozoites	Human Lf (2mg/ml) (Kassim et al., 2000)	78%				
	Lf (50µg/ml) (Parra et al., 2013)	95%				
***Toxoplasma*** Trophozoite	bLf apo/mono (20µg/ml) (Anand et al., 2015b)	20%				
	C peptide and BLf (1mg/ml) (Tanaka et al., 1995)	80%	Lfcin B (1mg/ml) In vitro and in vivo mouse model	0% (Tanaka et al., 1995)	Alginate chitosan calcium phosphate bovine Lactoferrin nanoparticles	1.2% (w/v)(Anand et al., 2015b)
	Lfcin (1mg/ml)	0%				
	Human Holo (Tanaka et al., 1997)	55%				
	Lf (1mg/ml) (Dzitko et al., 2007)	65%				
***Trypanosoma*** amastigotes	Human apo Lf (10µg/ml) (Lima and Kierszenbaum 1987)	54%	–	–	–	–
***Babesei*** merozoites	bLf apo (2.7mg/ml) (Ikadai et al., 2005)	50%	–	–	–	–

### 3.1 Role of Lf against *Leishmania*

Leishmaniasis is a disease that is caused by the protozoan parasite *Leishmania* and it affects approximately 13 million people annually (Kevric et al., 2015). The parasite alters between promastigote (insect host) and amastigote stages (mammalian host). There are three different forms of leishmaniasis: visceral, cutaneous, and mucocutaneous. Visceral leishmaniasis is caused by *L. donovani, L. chagasi*, and *L. infantum*. Cutaneous leishmaniasis is caused by *L. brazilensis* and *L. (L.) amazonensis*, and mucocutaneous leishmaniasis is caused by *L. guyanensis* and *L. panamensis* (Palumbo, 2010; Reimão et al., 2020). The treatment for the disease is pentavalent antimonial compounds, but due to the emerging resistance, these drugs are not effective (Sundar et al., 2000; Maertens et al., 2022). However, the most recent and effective therapy against leishmaniasis is amphotericin B and its liposomal nanoformulations, but due to the high cost of the drug, and toxicity, its usage is limited (Saravolatz et al., 2006; Maertens et al., 2022). The antileishmanial activity of Lf has been rarely studied; however, some reports have manifested the acquisition of radiolabeled iron by the promastigote form of *L. chagasi* through *in vitro* studies describing the role of a common receptor or convergent pathway. The promastigote form of the parasite has been known to acquire iron from various sources, which include Lf, Tf, and hemin. This study describes the uptake of the reduced form of iron (Fe^2+^) instead of the oxidized form (Fe^3+^) derived from Lf protein by the promastigotes (Wilson et al., 1994). These results suggested that the promastigote form of the parasite may have a different pathway of internalization, and based on that, the parasite may show different aspects of pathogenicity and survival.

### 3.2 Potent activity of Lf peptides against *Leishmania*

Potent Lf AMPs, namely, Lfchimera, Lfcin, and Lfamphin, were found to be leishmanicidal in nature when evaluated through an *in vitro* study (**Table 2**) (Silva et al., 2012). These peptides showed internalization and accumulation inside the cell, leading to impaired ATP production, and loss of ionic gradient, resulting in parasite death. The activity of Lfchimera has largely surpassed the other two derivatives as it appears to attach the membrane of the parasite leading to its destruction. The role of either the Lf native protein form or its various derivatives has suggested the destruction of the parasite through imbalance in the gradient ion channel, which might have changed the electrolyte balance and osmoregulation (Silva et al., 2012).

### 3.3 Lactoferrin nanotherapy used against *Leishmania*

There are several natural biological molecules that are used as a loading drug in nanotherapy. Lf nanoformulation has been studied against a wide range of diseases such as cancer and parasitic infection (Kondapi, 2020; Varma et al., 2020). Previously, the antileishmanial activity of Lf-appended amphotericin B bearing nano reservoirs has been thoroughly addressed against this parasite using *in vitro* and *in vivo* studies, showing reduced parasite burden (Asthana et al., 2015). The nanoparticles were taken up more efficiently by the host macrophages and suggested their targeted delivery. This nanoformulation induced a high Th1 immune response in mice that contributed to reduced parasite burden in the visceral organs with low toxicity (Asthana et al., 2015). Another study showed the antileishmanial activity of the modified polylactic-co-glycolic acid (PLGA) nanoparticle (NP) incorporated with Lf protein. These nanoparticles were loaded with betulinic acid (BA), which is a naturally occurring triterpenoid and has shown promising antitumor and anti-inflammatory properties (Halder et al., 2018). The mechanism of action of BA has been found to be through the destruction of mitochondrial membrane potential and ROS production (Cichewicz and Kouzi, 2004). However, BA has poor solubility, but its loading with PLGA nanoformulation has been found to enhance its targeted delivery. Moreover, author found the incorporation of Lf protein helped in the receptor-mediated binding of these NPs and improved its further engulfment inside the cells. With these modifications, Lf-embedded BANP showed a significant reduction in intracellular amastigotes through high production of NO and enhanced Th1 response through elevated levels of IL-12 and reduced expression of IL-10 in host macrophages (Halder et al., 2018).

### 3.4 Antiparasitic activity of apo Lf against *Plasmodium*

*Plasmodium* is an intracellular protozoan parasite known to cause malaria, which leads to the mortality of 600,000 people annually (WHO, 2022). The most deadly form of the disease is called falciparum malaria, which is caused by, *P. falciparum*, responsible for almost 90% of malaria cases alone in Africa (Snow et al., 2005). The *Plasmodium* parasite completes its life cycle in two different hosts, an invertebrate host (mosquito) and a vertebrate host (human). The sporozoite stage of the parasite is transmitted through the mosquito bite to the human host and multiplies inside the liver cells. This sporozoite stage leads to the formation of merozoite, which transmits into the bloodstream and multiplies inside the RBCs. To avoid this transmission cycle, various transmission-blocking vaccines have also been under development (Buchholz et al., 2011; Nikolaeva et al., 2015). The current treatment strategy for malaria includes artemisinin therapy. Artemisinin and its derivates in combination with other drugs have also shown promising results against malaria (Abumsimir and Al-Qaisi, 2023). The parasite resides in the RBCs and feeds on the iron-containing Hb as it develops from the ring to the schizont stage and utilizes all the Hb iron as its nutrient (Egan et al., 2002). Therefore, iron chelators have been studied against *P. falciparum* as antimalarial agents for several decades (Thuma et al., 1998). Earliest reports have shown the effect of iron chelators like desferriferrithiocin (DFT), desferricrocin (DFC), and hLf on the *in vitro* growth of *P. falciparum.* The apo form of hLf showed growth arrest at the ring form of the parasite and inhibited its further conversion to the trophozoite stage, whereas no effect was observed after treatment with iron-saturated hLf (Fritsch et al., 1987). Pretreatment of Lf with the circumsporozoite (CS) protein of malarial sporozoites has shown interference in its attachment and invasion to liver cells, disrupting attachment with heparin sulfate (HS) and LDL receptor-related protein (LRP) (Shakibaei and Frevert, 1996; Sinnis et al., 1996). Lf has also shown anti-adherent properties. Lf-treated infected RBCs showed hindrance in their attachment to various receptors present on placenta and endothelial cells, namely, CD36 (Eda et al., 1999), intercellular cell adhesion molecule (ICAM-1), thrombospondin (TSP) (Roberts et al., 1985; Berendt et al., 1989), chondroitin sulfate A (CSA) (Robert et al., 1995; Fried and Duffy, 1996), and vascular cell adhesion molecule-1 (VCAM-1) (Ockenhouse et al., 1992).

Previous clinical studies have suggested the role of Lf protein as a major component of human milk in providing protection to the fetus against malaria. Breast milk has been found to be high in IgM and IgA, and the role of Lf in protecting the mother and the developing fetus through vertical transmission has been suggested (Kassim et al., 2000). Another study postulated that BCG immunization in mice led to increased concentration of Lf protein in the plasma and helped reduce *P. yoelli* infection in mice along with an increased number of CD4 and CD8 cells (Parra et al., 2013). These results suggest the immunomodulatory action of Lf protein. Previous studies have explored the role of Lf protein in reducing the invasion capacity of parasitized erythrocytes inside the new RBCs. Reduced invasion of RBCs leads to a significant reduction in parasite levels, by 71% and 95% at day 14 and 17 post-infection (poi) compared to the untreated group. This reduction in parasite levels was attributed to the presence of Lf as a major factor and was found to be independent of antibody production (Parra et al., 2013). All these studies have pointed out the role of Lf in inhibiting malarial parasites, but a detailed mode of action was not fully demonstrated.

The studies performed using Lf as an antimalarial agent did not fully illustrate its effect on the RBCs; therefore, it is of utmost importance to understand its cytotoxicity and mode of action against malarial parasites. A previous extensive study conducted by our group elaborated on the interaction of RBCs with various iron-saturated forms of bLf and buffalo Lf (buLf) (Lfs), such as the apo, mono, and holo form (Anand et al., 2015a). Different concentrations of these two forms of Lf and different iron saturation were examined against the human RBCs and effect on its shape and size. All different forms of Lfs were found to be nontoxic in nature and did not show any alteration in the size and shape of RBCs when given in a dose- and time-dependent manner. RBCs treated with holo Lfs showed high production of ROS as compared to apo/mono forms of the Lfs (Anand et al., 2015a). After analyzing the nontoxic nature of various Lfs toward RBCs, we intended to study its effect on intracellular parasites, and we chose to work with buLf protein in mouse models, which showed better effect over bLf.

### 3.5 Antimalarial activity of Lf in its native and nanoformulation form

Our group has studied the role of buLf protein in its native form and nanoformulation against *P. berghei*-infected mice. Previous studies from our group have suggested that Lf protein works best when it is being administered orally (Kanwar et al., 2012). However, Lf is a cationic protein and can degrade through the gastric pH and enzymatic activity when incorporated via the oral route of administration. Therefore, our group has coupled this protein with biodegradable polymers like alginate and chitosan to make a nontoxic bioactive drug and studied its activity against colon cancer and further against the *P. berghei* mouse model (Kanwar et al., 2012; Anand et al., 2016). A significant reduction in parasite load was observed when infected BALB/c mice were treated with the buLf mono form. However, enhanced antiparasitic activity was seen after giving its nanoformulation using alginate and chitosan nanocarriers (buLf NC) through oral administration (Anand et al., 2016). Mice treated with either native buLf or buLf NC did not show any pathophysiological alteration and had significantly less peripheral parasitemia and high ROS production in spleen and liver cells when compared to the untreated group. Biodistribution of buLf in the liver and spleen showed its entrapment in liver Kupffer cells and the red pulp of the spleen. Moreover, the treatment with either native buLf or buLF NC resulted in maintaining the iron levels and iron metabolism in mice when compared with control mice, which were found to be anemic. The expression for miRNAs of iron metabolism, such as miR-Let7d, miR122, miR-196 miR-200b, miR-210, miR-214, miR320, miR-485 and miR-584, was found to be elevated in mice which showed balanced iron metabolism (Anand et al., 2016). Therefore, our study suggests the chelating effect of Lf protein and the targeted delivery of Lf protein inside the macrophages, which help prevent parasite multiplication and help maintain iron metabolism.

### 3.6 The promising antiparasitic activity of apo/mono Lf and its peptide against toxoplasmosis

*Toxoplasma gondii* is an obligate parasite known to cause toxoplasmosis in humans and immunocompromised patients. It is generally assumed that approximately 25% to 30% of the world’s human population is infected by *Toxoplasma* and approximately 500 million humans have antibodies to this parasite (Ajioka and Morrissette, 2009). *T. gondii* mice model has been well studied before and known to cause enchephalitis. Various reports have studied the chronic infection of *T. gondii* in mice previously which causes the formation of brain cyst. These mice mimic the ideal pathological conditions as with humans and shows cytokine response that can be picturalizsed in human in order to study the pathophysiology of the diseases (Lutshumba et al., 2020; Anand et al., 2022). Conventional drugs used for the treatment of toxoplasmosis are sulfadiazine and pyrimethamine, but resistance against sulfadiazine has been emerging for the last few years (Doliwa et al., 2013). The efficacy of Lf protein studied against *Toxoplasma* dates back to 1995, when bLf and its peptides, namely, the Lfcin B and C-terminal peptide, were used to assess their antitoxoplasmal activity. The parasite was incubated with the bLf and its various peptides and studied for its invasive tendency. Interestingly, the Lfcin B peptide significantly hindered the invasive capacity of tachyzoites to infect the cells when compared to untreated control parasites and the C-terminal peptide. The authors suggested that although the tachyzoites were alive after the treatment with Lfcin B, they possibly lost the capability to invade the cells (Tanaka et al., 1995). This effect was further studied in a mouse model, wherein all mice treated with Lfcin B survived until day 30 post-injection as compared to untreated mice, which died by day 9 post-infection (Tanaka et al., 1995). These results suggest that the Lf peptide diminishes the invasion capacity of tachyzoites in *in vitro* and *in vivo* models. The authors further investigated a number of studies to identify the mode of action of Lf protein. The authors intended to study the pretreatment effect of Lf on murine somatic host cells and revealed a reduction in the number of intracellular tachyzoites invading when seen in comparison to the untreated control group (Tanaka et al., 1996). Furthermore, the authors tried to subject intracellular tachyzoites to different iron-saturated forms of Lf, and it was observed that only apo/mono bLf forms showed the best inhibitory activity with increased free radical ion production (Tanaka et al., 1997). Similar results were obtained when the antiparasitic activity of bLf was observed in mice that were orally administered with the protein after infection with *T. gondii.* Treatment results suggest reduced parasite load through the production of intracellular ROS and NO (Tanaka et al., 1997). These studies suggested iron chelation and ROS production as potential mechanisms of action of bLf. However, the authors discovered the possible role of tyrosine kinase phosphorylation inside the cells to be the mode of action of Lf effect, along with ROS production (Tanaka et al., 1998).

The effect of the holo form of hLf was observed against intracellular tachyzoites and monitored; it did show inhibition when used in a dose- and time-dependent manner, but no remarkable effect was found on the extracellular stages of the parasite (Dzitko et al., 2007). This study correlates with the finding that the iron-saturated form of Lf protein can induce ROS production and kill the parasite, whereas the apo/mono form can inhibit the extracellular stages. Another study has documented the role of parasite rhoptry proteins, namely, ROP2 and ROP4, as important genes responsible for the invasion and pathogenesis of the parasite (Carruthers, 1999). However, these proteins have been reported to acquire iron molecules from hLf, helping the attachment of tachyzoites with cell surface-promoting parasite multiplication and enhanced pathogenicity (Dzitko et al., 2007).

To detail the interaction between the parasite and the macrophage host cells, our group has performed a multiparametric study to examine the same (Anand et al., 2015a). Our study demonstrated the effect of various iron saturations of bLf and buLf on morphology, cytotoxicity, the production of free radical ions, and the phagocytic property of macrophages using the THP1 cell line. After the treatment of the holo form of Lf, the macrophages showed increased ROS and phagocytic properties with no cytotoxicity and morphometric alterations (Anand et al., 2015a). We studied a similar effect on macrophages that were infected with tachyzoites of the parasite. Infected macrophages were incubated with different concentrations of apo/mono Lf and the treatment showed reduced number of infected macrophages when compared with holo Lf. However, when the intracellular parasites were counted per macrophage, again, the apo/mono form of the bLf showed both a significantly smaller number of infected macrophages and a smaller number of intracellular tachyzoites per macrophage (Anand et al., 2015b).

### 3.7 Lactoferrin nanotherapy used against *Toxoplasma*

In our previous study we investigated the role of bLf on acute stage of the mice. The *in vivo* activity of mono bLf and its nanoformulation using alginate chitosan bLf NC were analyzed by our group (Anand et al., 2015b). The bLf NC showed better antiparasitic activity in comparison to mono bLf protein in reducing the parasite load and provided a high Th1 immune response, resulting in the prolonged survival of mice. We also found cyst development inside the liver tissue of the mouse which were treated with bLf and bLf NC. These results showed the treatment effect of Lf protein on the parasite which lead it to the cyst formation. Our study proposed that bLf NC treatment induces increased ROS production in the liver and spleen, followed by high NO production. With the help of immunohistochemistry, we observed immunoreactivity of Lf in the spleen and liver, suggesting the targeted delivery of Lf in respective visceral organs with very low toxicity. Also the biodistribution of Lf protein was found in the liver of the mice. The proposed mechanism of action from our study suggested that the increased production of ROS and NO caused intracellular parasite killing. Additionally, Lf caused potential disruption of iron availability for parasite survival and maintained iron metabolism, leading to improved protection against parasitic infections in mouse models (Anand et al., 2015b).

### 3.8 Antiparasitic activity of Lf against *Trypanosoma*

*Trypanosoma* is a blood-borne parasite that causes sleeping sickness, also known as African trypanosomiasis or Chagas disease in humans. The incidence rate of the disease may be as high as 90% in endemic areas, especially in Africa (Büscher et al., 2017). Conventional drugs such as suramin, pentamidine, and melarsoprol are the treatment of choice (Garcia-Salcedo et al., 2016). The parasite multiplies in the reticuloendothelial system (RES) of humans as well as in muscle cells. It relies on the source of iron for its multiplication and for its conversion from one form to another (Stijlemans et al., 2015, 2018). Previous studies have suggested the correlation of iron content in a patient’s serum with the severity of trypanosomiasis (Murray et al., 1978). Many studies have investigated the potential of Lf against *Trypanosoma* parasite due to its structural similarity with *Leishmania* parasite. An earlier study examined the effect of Lf protein on intracellular amastigotes’ growth using different host cells, namely, mouse peritoneal macrophages (MPMs) and human blood monocytes (HBMs) (Lima and Kierszenbaum, 1987). The inhibitory effect of Lf was found to be more pronounced in MPM cells than in HBM cells. The rationale for the difference in inhibitory activity was correlated with the higher number of Lf receptors present on the surface of MPM cells, which may facilitate the entry of Lf inside the cell and show an inhibition effect (Lima and Kierszenbaum, 1987). Another study showed that Lf can serve as a cell surface marker on the amastigote form of *T. cruzi*, resulting in opsonization, to inhibit the parasite load in host cells such as monocytes and macrophages (Lima et al., 1988). A study conducted by Tanaka et al. suggested that bLf binds to a particular surface protein of 40 kDa, identified as Glyceraldehyde 3 phosphate (G3Ph) through receptor ligand binding and suggested a possible correlation with parasite inhibition (Tanaka et al., 2004).

### 3.9 Antiparasitic effect of native apo Lf protein against *Babesia cabelii*

Babesiosis is a disease caused by the protozoon blood-borne parasite *Babesia*. It has similar morphology and pathogenicity to that of the *Plasmodium* genus; however, the two parasites differ in their mortality rate (Allred and Al-Khedery, 2004; Pritt, 2015). *Plasmodium* is responsible for almost 600,000 deaths annually, whereas *Babesia* has a low incidence rate, which has been reported only in North America, Japan, Korea, Taiwan, and India (Vannier and Krause, 2012; Onyiche et al., 2021). In North America alone, a total of 50,856 cases were found in 2019 (Swanson et al., 2023). Like *Plasmodium*, *Babesia* also feeds on Hb of the RBCs and hunts for iron; few studies have investigated the role of Lf against this parasite. A previous study suggested the effect of different iron-saturated forms of bLf such as apo, holo, and mono Lf, and the peptide LfH against two species of *Babesia* such as *B. cabelli* and *B. equi*, but only the apo form had shown inhibition toward *B. cabelli* and not against *B. equi* (Ikadai et al., 2005). The mode of action of Lf against *Babesia* was found to be similar to that of *Plasmodium*, i.e., through iron chelation, but still, *in vivo* studies are required to justify the antiparasitic nature with a detailed mode of action.

The mechanism of action summarized from the above-mentioned studies on these parasites demonstrated the iron-chelating effect of apo Lf from the parasite through receptor-mediated binding, whereas the holo form of Lf has provided growth and helped enhance the pathogenesis of the parasite through surface internalization. However, in some intracellular parasites such as *Plasmodium, Toxoplasma*, and *Leishmania*, holo Lf may act through the production of free radical ions, reactive oxygen species (ROS), and nitric oxide (NO), which results in the intracellular killing of the parasite (**Figure 1**).

## 4 Effect of Lf protein on extracellular parasites

Lf protein isolated from bovine or human milk has also shown inhibition against different extracellular parasites, and the mechanism of action is fairly similar to that of intracellular parasites.

### 4.1 Role of Lf against *Acanthamoeba*

*Acanthamoeba* is a free-living parasite that can cause opportunistic infections in humans and has an estimated incidence rate of 1.2 per million adults. The parasite is known to invade host cells and acquire iron for its pathogenesis, causing keratitis and corneal infections (Page and Mathers, 2013; Niederkorn, 2021). Previous research has shown that *A. castellanii* proteases play a role in acquiring iron from holo hLf and holo Tf, which helps in its multiplication and pathogenesis (Ramírez-Rico et al., 2015), whereas the apo bLf has been reported to show amoebicidal effect against the trophozoite form of *A. keratitis* (Tomita et al., 2016). After the apo Lf treatment, the trophozoite shape was found to be globulus as compared to the normal non-globulus form of the trophozoites. However, the study did not report any notable effect on the cystic stage of the parasite, but the treatment with apo Lf prevented the conversion of trophozoites to cysts. This could result from the inability of Lf to penetrate the cell wall of the parasite (Alsam et al., 2008). A recent study by Ramírez-Rico et al., (2023) conducted similar experiments using apo-bLf protein on *A. castellanii*, which causes granulomatous amoebic encephalitis and keratitis in humans. The authors did not see any amoebicidal effect on the parasite trophozoite form and showed resistance in a dose- and time-dependent manner. The cytopathic effect was observed on *A. castellanii* when preincubated with apo-bLf. The cytopathic effect was found to be diminished after the apo-bLf treatment, whereas its absence caused damage to the parasite. Authors suggested the role of cysteine and serine proteases in the pathogenesis of the amoeba, which showed a cytopathic effect (Ramírez-Rico et al., 2023).

### 4.2 Role of Lf against *Trichomonas*

*Trichomonas vaginalis* is a parasite that causes sexually transmitted disease (STD) in men and women, affecting 275 million cases annually, but most of them remain asymptomatic (Johnston and Mabey, 2008; Kissinger et al., 2022). Parasites reside in flagellated trophozoites inside the female genital tract and replicate by binary fission (Yadav, 2023). Metronidazole and tinidazole are the drugs of choice for the treatment of *T. vaginalis* infections (Rigo et al., 2022). Several studies have identified iron as an essential micronutrient for the virulence and pathogenicity of *Trichomonas* (Song, 2016; Rivera-Rivas et al., 2020). The first interactive study of *Trichomonas* with Lf described how the parasite takes up the iron from the iron pool of the plasma membrane of the cells. The parasite sequesters the iron from hLf through receptor-mediated binding and its accumulation inside the trophozoite resulted in its increased pathogenesis. The enzymatic activity of pyruvate/ferredoxin oxidoreductase was suggested to be responsible for the binding of hLf to the parasite as it helps with iron acquisition (Peterson and Alderete, 1984).

Another previous study has suggested that the synergistic effect of complement C3 with Lf protein causes cell lysis of *T. vaginalis*. This study demonstrated that the presence of iron in the parasite cultivation media sourced from either Lf or Tf showed an inhibitory effect. However, in the absence of an iron source, the antiparasitic effect predominantly came from complement-mediated lysis (Alderete et al., 1995). *Trichomonas* is known to secrete a number of cysteine proteases, collectively called cysteine proteases 30 (CP30), which have shown an apoptotic effect on the epithelial cells of the host (Kummer et al., 2008). The authors reported that the presence of iron sourced from holo Lf in cell culture media leads to the reduced activity of CP30 and the survival of the parasite. However, in the absence of an iron source, the CP30 enzyme performed the proteolytic function and damages the epithelial cells (Kummer et al., 2008). Thus, these studies signify the role of Lf in promoting the growth of *Trichomonas* by stimulating different signaling pathways, resulting in its pathogenesis.

## 5 Discussion

Lf research has progressed over several decades, expanding its utility in various health-related areas. It is mentioned as a growth supplement in infant formulas and for its role in iron metabolism. Lf protein has shown its efficacy not only by chelating iron but also through its binding efficacy to the DNA molecule. Lf protein has a DNA binding site, which makes this protein more crucial in targeting or activating different transcription factors responsible for various cellular or biochemical activities (Fleet, 1995). It has the ability to provide adaptive and innate immunity to fight various diseases. Lf is extracted from bovine, sheep, or goat milk. Over decades, there have been improvements in the production of Lf. This protein is able to express in many microbes such as *Escherichia coli* and some fungi such as *Aspergillus oryzae* and *Pichia pastoris*, and its production has been improved through recombinant technology. Studies have also used CHO cell lines for the expression of this protein, and other cell-free protein methods have also been successful in its production. There has been a significant improvement in the yield of Lf after producing a transgenic fetal calf that can express this protein and enhance the yield.

Overall, this review suggests that the Lf protein exerts its effect on human parasites through iron chelation and free radical ion production where other possible mechanisms of action may also play a role. Parasites gain their nutrition and enhance their virulence by withdrawing the iron molecule from host cells or its surrounding. Treatment with the low-iron form of the Lf protein, such as the apo or mono form, can chelate the surrounding iron and prevent the iron from being available to the parasite. More specific binding of Lf protein with the target protein site of the parasite can be achieved by Lf peptide formulation, which can show a more specific and targeted killing effect with minimum toxicity. Nanoformulation of Lf protein is another strategy for delivering this drug. Nanoformulation of the Lf drug has been suggested because it is easy to incorporate with a variety of particles and targeted drug delivery. This review not only covers the antiparasitic nature of Lf but also highlights the immunomodulatory effect of this drug. Lf research has advanced over several decades and has expanded this protein’s value and importance in various health-related areas as a growth booster and for iron metabolism. Many countries are considering using Lf protein in clinical trials as a growth and immune supplement. Therefore, this review emphasizes the need for ongoing research to better understand the role of Lf and its mechanism of action, especially in the context of emerging infectious diseases. In-depth studies are thus required to provide possible insights into how Lf can be optimally utilized for parasitic infections.

## Author contributions

NA: Conceptualization, Writing – original draft, Writing – review & editing.
